# Identification of genetic markers associated with coronary artery disease in Pakistani Pashtun population: A case–control association study

**DOI:** 10.1097/MD.0000000000046983

**Published:** 2026-01-02

**Authors:** Asif Jan, Aftab Ullah, Mashal Khattak, Waheed Ali Shah, Rani Akbar, Ursula Abu Nahla

**Affiliations:** a Saidu Group of Teaching Hospital, Saidu Sharif Swat, KP, Pakistan; b District Headquarter Hospital Charsadda, KP, Pakistan; c Department of Pharmacy, University of Peshawar, KP, Pakistan; d Department of Pharmacy, Abdul Wali khan University Mardan, KP, Pakistan; e Faculty of Medicine, Hebron University, Hebron, Palestine.

**Keywords:** coronary artery disease, genetics, genetics and genomics of cardiovascular disease, Pakistan, Pashtun, polymorphism, population

## Abstract

Genetic makers play a critical role in coronary artery disease (CAD) susceptibility. This study investigates the association between angiotensin-converting enzyme (ACE) insertion/deletion (I/D) and endothelial nitric oxide synthase (eNOS)-786 T > C polymorphism with CAD in the Pashtun ethnic population of Khyber Pakhtunkhwa, Pakistan. This case–control study was conducted on 1000 individuals, including 500 CAD patients and 500 healthy controls. Genotyping of ACE I/D and eNOS-786 polymorphisms was performed using polymerase chain reaction and polymerase chain reaction-restriction fragment length polymorphism techniques. The association of polymorphisms with CAD risk was analyzed using logistic regression to determine odds ratios (ORs) with 95% confidence intervals (CIs). The ACE deletion (D) allele was significantly more frequent in CAD patients than in controls (OR = 4.76, 95% CI: 3.21–7.08, *P* < .001), indicating a strong genetic predisposition. Similarly, individuals with the ACE DD genotype had a markedly higher risk of CAD (OR = 6.42, 95% CI: 3.89–10.59, *P* < .001) compared to individuals with II genotype. In contrast, no significant association was observed between eNOS-786 polymorphism and CAD risk (OR = 1.08, 95% CI: 0.72–1.61, *P* = .98). The ACE I/D polymorphism, particularly the DD genotype, is strongly associated with an increased risk of CAD in the Pashtun population, while eNOS-786 polymorphism does not appear to be a significant risk factor. These findings underscore the importance of genetic screening for early risk assessment and developing intervention therapies according to genetic make-up of individuals for more effective disease prevention.

## 1. Introduction

Cardiovascular diseases (CVDs) remain one of the leading cause of morbidity and mortality worldwide, accounting for nearly 17.9 million deaths annually.^[1]^ CVDs has a profound impact on multiple aspects of health, including physical, cognitive, and psychosocial well-being. Among various forms of CVDs, coronary artery disease (CAD) is particularly significant, as it leads to myocardial infarction, heart failure, and other severe complications. Genetic predisposition, in conjunction with environmental and lifestyle factors, plays a critical role in the pathophysiology of CAD.^[2]^ Understanding the genetic risk factors contributing to CAD is essential for early detection, prevention, and targeted therapeutic interventions. The renin–angiotensin system (RAS) is a crucial regulator of blood pressure and cardiovascular homeostasis. Genetic polymorphisms within key genes of the RAS, such as the angiotensin-converting enzyme (ACE) gene confer risk for CAD. The insertion/deletion (I/D) polymorphism in the ACE gene has been implicated in increased cardiovascular risk by modulating angiotensin II levels, thereby influencing vascular function, inflammation, and thrombosis.^[3,4]^ Several studies have reported a strong association between the ACE D allele and the development of CAD, particularly in populations with high genetic homogeneity.^[5]^

Another critical regulator of vascular function is nitric oxide (NO), which is synthesized by endothelial nitric oxide synthase (eNOS). NO plays a protective role in the cardiovascular system by promoting vasodilation, inhibiting platelet aggregation, and reducing oxidative stress.^[6]^ The eNOS gene has various polymorphic variants, including the eNOS-786C/T polymorphism, which has been investigated for its potential role in vascular dysfunction and CAD risk. However, conflicting results have been reported across different populations, indicating the need for population-specific genetic studies.^[7]^ The Pashtun population of Khyber Pakhtunkhwa, Pakistan, presents a unique genetic background due to its distinct ancestry and consanguineous marriage patterns, which may contribute to an increased prevalence of hereditary diseases, including CAD. Despite the high burden of CVD like CAD in this region, there is a lack of genetic association studies examining the role of RAS and eNOS polymorphisms in this ethnic group.^[8]^ Identifying genetic risk factors specific to the Pashtun population can facilitate precision medicine approaches and improve cardiovascular risk assessment.

Previous research has demonstrated significant variability in the prevalence of ACE I/D and eNOS polymorphisms across different ethnicities. While some studies have found a strong correlation between the ACE D allele and CAD risk in South Asian and Middle Eastern populations, others have failed to establish such associations in European and East Asian cohorts.^[9,10]^ Similarly, the impact of eNOS polymorphisms on vascular function and CAD remains inconclusive, with some studies indicating a protective effect of the T allele, while others report an increased risk associated with the same variant.^[11]^ These fluctuating results highlight the necessity of conducting population-specific genetic studies to elucidate the role of these polymorphisms in CAD pathogenesis. In this study, we aimed to investigate the association of ACE I/D and eNOS-786C/T polymorphisms with CAD in a cohort of 1000 Pashtun individuals from Khyber Pakhtunkhwa, comprising 500 CAD patients and 500 healthy controls. By analyzing these genetic markers, we seek to provide insights into the genetic predisposition to CAD in this population and contribute to the broader understanding of cardiovascular genetics. The findings of this study may have implications for early disease detection, risk stratification, and the development of targeted therapeutic strategies for high-risk individuals.

## 2. Materials and methods

### 2.1. Study design, setting and CAD population selection

This multi-center, case–control study was conducted across 3 major hospitals in Khyber Pakhtunkhwa, Pakistan; Lady Reading Hospital, Peshawar; Mardan Medical Complex, Mardan and DHQ Hospital, Charsadda. A total of 1000 individuals of Pashtun ethnicity were included in this case–control study. The study participants were grouped as: control individuals (having no CAD, n = 500) and cases/patients (individuals with CAD, n = 500). Control individuals were defined as those who did not experience or have CAD, whereas patients were those suffering from CAD. Patients with CAD were from different districts including Swat, Dir, Kohat, Charsadda, Bannu, Mardan, Nowshera, Swabi, and Peshawar. The study participants were registered at cardiac care units of the above mentioned. These hospitals have separate state of the art setting where patients with CVDs are treated. Agreement to participate in the study was collected/obtained from all individuals who participated in this study. For easy understanding of uneducated patients, the agreement form or consent form was verbally explained in the local Pashto language. Once they agreed to take part in the study, they either signed the form themselves or had an attendant or relative sign on their behalf. Individuals with any form of vascular disease (including peripheral arterial disease and cerebrovascular events), malignancy, or chronic inflammatory and autoimmune disorders were systematically excluded from both case and control groups.

### 2.2. Control subjects selection and exclusion criteria

Control subjects were recruited from outpatient clinics and community screening camps. They were clinically assessed to be free from CAD and other vascular or chronic inflammatory diseases using ECG and physician evaluations. Controls were matched for age and sex, and conditions like hypertension and diabetes were not overrepresented to avoid exposure-based bias.

### 2.3. Ethics committee approval

This research was conducted in accordance with internationally accepted ethical standards for biomedical research involving human participants. The study protocol was reviewed and approved by the Research and Ethical Committee of District Headquarter (DHQ) Hospital Charsadda, Khyber Pakhtunkhwa, Pakistan (Approval No. 293/MSDHQ, dated November 01, 2024). All procedures were carried out in compliance with the ethical principles outlined in the Declaration of Helsinki (2013), ensuring respect for human dignity, safety, and rights. Informed consent was obtained from all study participants prior to sample collection, and confidentiality of participants’ data was strictly maintained.

### 2.4. Collection of demographic and clinical data

A structured proforma, carefully designed for this study was used to collect both demographic and clinical details of the participants. The gathered information covered various aspects, including gender, age, and district of residence. Additionally, data on lifestyle factors such as smoking habits and exercise routines were recorded. The presence or absence of comorbid conditions was also documented. Furthermore, socioeconomic background, adherence to prescribed medications, and dietary compliance were assessed to obtain a comprehensive understanding of each participant’s health profile. Out of 1100 individuals approached, 1000 participated. Of these, 100 were excluded based on predefined exclusion criteria (e.g., presence of vascular, malignant, or inflammatory conditions), or due to incomplete clinical data. Less than 5% refused to become part of the study, primarily because of family issues/personal issues, and some does not participate because they were not local residents. To reduce response bias, data was corroborated with medical records whenever feasible.

### 2.5. Collection of samples and genetic analysis

Blood samples were collected and analyzed as described previously.^[12]^ Briefly venous 5 mL of venous blood was collected from each participant in EDTA-containing tubes. Genomic DNA was extracted using kit method and quantified using a Nano-Drop spectrophotometer. Polymerase chain reaction (PCR) and PCR-restriction fragment length polymorphism assays were used for genotyping the ACE I/D and eNOS-786C/T polymorphisms. Primer sequences were obtained using the National Centre for Biotechnology Information database. The selected primers were designed based on gene sequences relevant to the study, optimizing for parameters such as GC content, melting temperature, and amplicon size to ensure efficient PCR performance. All PCR reactions were carried out under optimized conditions. The amplified products were analyzed through gel electrophoresis on a 3% agarose gel to confirm genotype distribution. To ensure accuracy, 10% of samples were re-genotyped, showing 100% concordance. For ACE I/D, insertion-specific primers were used to counteract preferential D allele amplification. PCR reactions included positive and negative controls, and all gel results were independently verified by 2 trained technicians.

### 2.6. Statistical analysis

Data analysis was conducted using SPSS version 26.0.^[13]^ Descriptive statistics were presented as mean ± standard deviation for continuous variables and percentages for categorical data. Genotypic and allelic frequencies were compared between cases and controls using the chi-square test. The association between genetic polymorphisms and CAD risk was determined using logistic regression, adjusted for potential confounders, and presented as odds ratios (ORs) with 95% confidence intervals (CIs). A *P*-value < .05 was considered statistically significant. Hardy–Weinberg Equilibrium (HWE) was assessed in the control group for both ACE and eNOS polymorphisms. Logistic regression models were rerun with adjustment for age, gender, BMI, hypertension, diabetes, and hyperlipidemia to control for confounding.

## 3. Results

### 3.1. Description of the study cohort

Detail sociodemographic characteristics and co-morbidities prevalence in the study participants (n = 1000) are given in Tables 1 and 2. Within the control group, males were in majority, making up 75.5% of the participants, while females accounted for 24.5%. When considering the participants’ district of residence, 25.5% were from Peshawar, followed by 22.5% from Charsadda and 17.5% from Nowshera, with the remaining individuals distributed across other districts (see Table 1 for details). Regarding occupation, farming was the most common profession, with 27.5% of the participants engaged in agricultural work. A notable 85.0% reported a family history of hypertension, whereas 15.0% did not have such a history. In terms of smoking habits, 70% of individuals identified as smokers, while the remaining 30% were nonsmokers. Drug and dietary compliance were good in 71.0% of individuals, whereas 29.0% showed poor compliance. Regarding socioeconomic status, 76.5% belonged to average-income families, while 11.5% came from poor backgrounds. Among CAD patients, 72.5% were males and 27.5% were females. The majority belonged to middle-income families (66.0%), while 23.0% had poor financial backgrounds. Co-morbidities such as type 2 diabetes, kidney failure and hyperlipidemia were more prevalent in CAD patients compared to the control group.

**Table 1:** Sociodemographic features of the study subjects.

Variable	CAD patients (n = 500)	Healthy controls (n = 500)	*P*-value
Male (%)	72.5%	75.5%	.281
Female (%)	27.5%	24.5%	
Mean age (yr)	56 ± 13	54 ± 13	.705
Mean weight (kg)	62.6 ± 6.1	60.5 ± 8.3	.813
District of residence			.512
Peshawar	27.5%	25.5%	
Charsadda	17.0%	22.5%	
Mardan	11.0%	6.5%	
Kohat	6.0%	5.5%	
Swabi	9.5%	2.0%	
Nowshera	8.5%	17.5%	
Bannu	9.0%	5.0%	
Karak	2.5%	6.5%	
Dir	3.0%	5.0%	
Swat	6.0%	5.0%	
Occupation			
Business	10.0%	8.0%	.231
Govt Servant	18.5%	14.0%	
Retired	17.5%	15.0%	
Farming	17.5%	27.5%	
Housewife	20.0%	17.5%	
Labour	16.5%	18.0%	
Family history of hypertension			.031
Yes	67.5%	85.0%	
No	32.5%	15.0%	
Smoking status			.073
Yes	52.0%	70.0%	
No	48.0%	30.0%	
Drug and diet compliance			.043
Good	66.0%	71.0%	
Poor	23.0%	29.0%	
Socioeconomic status			.524
Good	11.0%	12.5%	
Average	66.0%	76.5%	
Below	23.0%	11.5%	

CAD = coronary artery disease.

**Table 2:** Prevalence of co-morbidities/ diseases other than CAD in study participants.

Comorbid condition	CAD patients (n = 500)	Healthy controls (n = 500)	*P*-value
Type 2 diabetes	110 (22.0%)	62 (12.5%)	.051
Hypertension	105 (21.0%)	95 (19.0%)	.611
Kidney failure	30 (6.0%)	20 (4.0%)	.912
Hyperlipidemia	115 (23.0%)	46 (9.1%)	.012
Hepatitis B (HBV)	0 (0.0%)	0 (0.0%)	–
Hepatitis C (HCV)	0 (0.0%)	0 (0.0%)	–

CAD = coronary artery disease.

### 3.2. Assessment of HWE

HWE analysis was performed for both ACE I/D and eNOS-786 (T > C) polymorphisms in the control group. The observed genotype distributions were compared with expected distributions using the chi-square (*χ*²) test. Both polymorphisms were found to be in HWE (*P* > .05), indicating no genotyping errors and random mating in the studied population. The details of HWE analysis are provided in Table 3. Moreover the study had sufficient statistical power to detect associations for the ACE I/D polymorphism. With 500 CAD cases and 500 controls, the calculated power exceeded 90% at α = 0.05 for detecting an odds ratio of 1.5 or higher, given the observed allele frequency differences between cases and controls.

**Table 3:** Hardy–Weinberg Equilibrium (HWE) analysis.

Gene	Observed genotype	Expected genotype	Chi-square (*χ*²)	*P*-value
ACE I/D	II:200, ID:200, DD:100	II:202.5, ID:195, DD:102.5	0.28	.59
eNOS-786	TT:435, TC:55, CC:10	TT:433.8, TC:56.4, CC:9.8	0.03	.86

ACE = angiotensin-converting enzyme, I/D = insertion/deletion, eNOS = endothelial nitric oxide synthase.

### 3.3. Genotypic and allelic distribution of ACE I/D polymorphism

**Figure 1.**
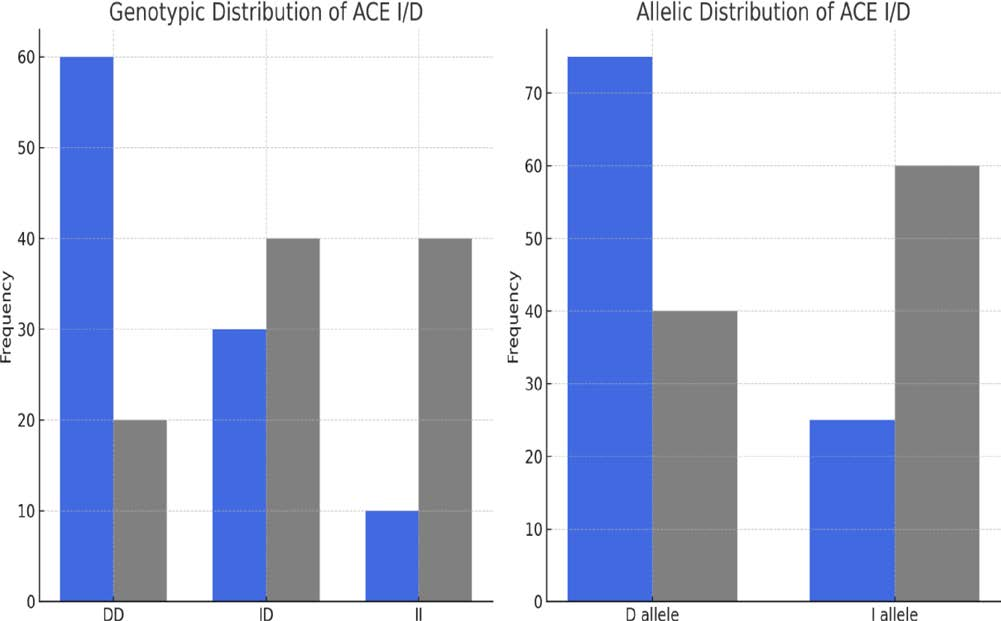
Genotypic/allelic distribution of ACE I/D in the study subjects. ACE = angiotensin-converting enzyme, I/D = insertion/deletion.

The frequency of the deletion (D) allele was significantly higher in CAD patients (75%) compared to controls (40%) (OR = 4.76, 95% CI: 3.21–7.08, *P* < .001). The DD genotype was present in 60% of the CAD patients compared to 20% of the controls, indicating a significant association with CAD risk (OR = 6.42, 95% CI: 3.89–10.59, *P* < .001). The ID genotype was observed in 30% of the cases and 40% of the controls, whereas the II genotype was significantly more frequent in controls (40%) than in cases (10%). Detail of genotypic and allelic distribution of ACE I/D is given in Figure 1 and Table 4.

**Table 4:** Genotypic and allelic distribution pattern of the ACE insertion/deletion (I/D) in study subjects.

Genotype/allele	CAD patients (n = 500)	Controls (n = 500)	OR (95% CI)	*P*-value
DD	300 (60%)	100 (20%)	6.42 (3.89–10.59)	<.001
ID	150 (30%)	200 (40%)	0.75 (0.56–1.02)	.07
II	50 (10%)	200 (40%)	Reference	–
D allele	750 (75%)	400 (40%)	4.76 (3.21–7.08)	<.001
I allele	250 (25%)	600 (60%)	Reference	–

ACE = angiotensin-converting enzyme, CAD = coronary artery disease, CIs = confidence intervals, ORs = odds ratios.

### 3.4. Genotypic and allelic distribution of eNOS-786 polymorphism

**Figure 2.**
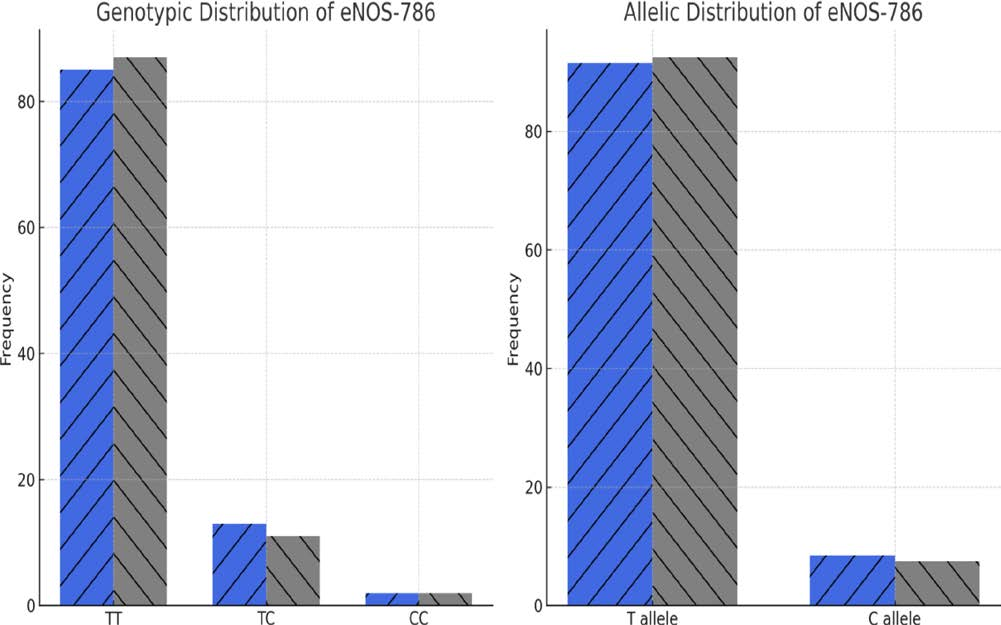
Genotypic/allelic distribution of eNOS-786 (T > C) in the study subjects. eNOS = endothelial nitric oxide synthase.

No significant difference was observed in the distribution of eNOS-786 polymorphism between cases and controls. The TT genotype was found in 85% of the CAD patients and 87% of the controls, while the TC genotype was observed in 13% of cases and 11% of controls. The CC genotype was rare, present in only 2% of both groups. The odds ratio for eNOS-786 polymorphism and CAD risk was 1.08 (95% CI: 0.72–1.61, *P* = .98), suggesting no significant association. Detail of genotype and allelic distribution of eNOS-786 polymorphism (T > C) is listed in Figure 2 and Table 5.

**Table 5:** Genotypic and allelic distribution pattern of eNOS-786 polymorphism in study subjects.

Genotype/allele	CAD patients (n = 500)	Controls (n = 500)	OR (95% CI)	*P*-value
TT	425 (85%)	435 (87%)	Reference	–
TC	65 (13%)	55 (11%)	1.08 (0.72–1.61)	.98
CC	10 (2%)	10 (2%)	1.00 (0.40–2.52)	.99
T allele	915 (91.5%)	925 (92.5%)	Reference	–
C allele	85 (8.5%)	75 (7.5%)	1.14 (0.78–1.67)	.67

CAD = coronary artery disease, CIs = confidence intervals, eNOS = endothelial nitric oxide synthase, ORs = odds ratios.

### 3.5. Association of ACE and eNOS polymorphisms with clinical parameters

**Figure 3.**
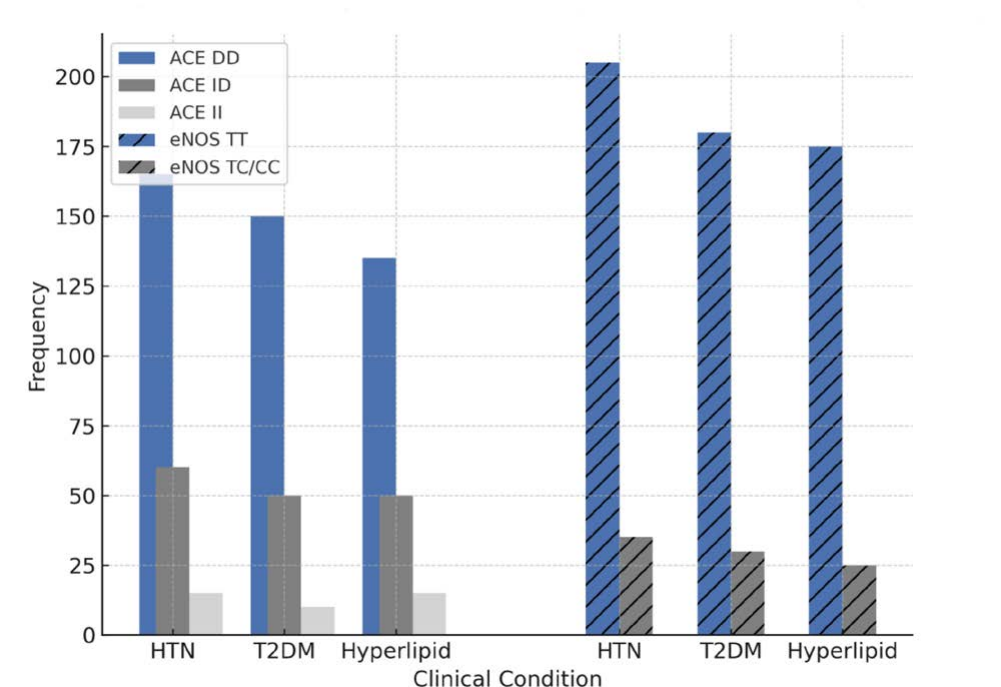
Association of ACE I/D and eNOS-786 (T > C) polymorphisms with clinical parameters in the study participants. ACE = angiotensin-converting enzyme, I/D = insertion/deletion, eNOS = endothelial nitric oxide synthase.

Among individuals with ACE DD genotype, a notable proportion exhibited cardiovascular risk factors, with 55% diagnosed with hypertension, 50% with Type 2 diabetes, and 45% with hyperlipidemia. This strong association suggests that the ACE DD genotype may contribute significantly to CAD susceptibility. In contrast, the eNOS-786 polymorphism showed no significant correlation with these clinical conditions, indicating that variations in this gene may not play a major role in the prevalence of hypertension, diabetes, or hyperlipidemia within the studied population. Details of Association of ACE and eNOS polymorphisms with clinical parameters is presented in Figure 3 and Tables 6 and 7. To investigate the confounding factors like age, sex, BMI, hypertension, diabetes, and hyperlipidemia affects on the association, we re-analyzed the data using multivariable logistic regression models adjusted for these covariates. The association of the ACE D allele and DD genotype with CAD remained statistically significant after adjustment, indicating that the observed effect is independent of these confounders. The lack of association for the eNOS-786 T > C variant also persisted in the adjusted model (Table 8).

**Table 6:** Association of ACE polymorphism with clinical parameters.

Clinical parameter	ACE DD (n = 300)	ACE ID (n = 150)	ACE II (n = 50)	OR (95% CI)	*P*-value
Hypertension	165 (55%)	60 (40%)	15 (30%)	1.95 (1.42–2.69)	<.001
Type 2 diabetes	150 (50%)	50 (33%)	10 (20%)	2.17 (1.53–3.07)	<.001
Hyperlipidemia	135 (45%)	50 (33%)	15 (30%)	1.82 (1.28–2.60)	.002

ACE = angiotensin-converting enzyme, CIs = confidence intervals, ORs = odds ratios.

**Table 7:** Association of eNOS polymorphism with clinical parameters.

Clinical parameter	eNOS TT (n = 425)	eNOS TC/CC (n = 75)	OR (95% CI)	*P*-value
Hypertension	205 (48%)	35 (47%)	1.02 (0.65–1.59)	.91
Type 2 diabetes	180 (42%)	30 (40%)	1.07 (0.68–1.70)	.78
Hyperlipidemia	175 (41%)	25 (33%)	1.38 (0.86–2.19)	.18

CIs = confidence intervals, eNOS = endothelial nitric oxide synthase, ORs = odds ratios.

**Table 8:** Adjusted association of ACE I/D and eNOS-786 T > C polymorphisms with CAD risk after controlling for classical risk factors.

Polymorphism	Genotype/allele	aOR (95% CI)	*P*-value
ACE I/D	DD vs II	5.97 (3.51–9.88)	<.001
	ID vs II	1.26 (0.80–1.99)	.315
	D allele vs I	4.51 (3.06–6.76)	<.001
eNOS-786 T > C	TC vs TT	1.10 (0.70–1.72)	.673
	CC vs TT	1.03 (0.39–2.67)	.951
	C allele vs T	1.12 (0.75–1.67)	.583

ACE = angiotensin-converting enzyme, CAD = coronary artery disease, CIs = confidence intervals, I/D = insertion/deletion, eNOS = endothelial nitric oxide synthase, ORs = odds ratios.

## 4. Discussion

CAD is polygenic disease influenced by both genetic and environmental factors. Among various genetic determinants, polymorphisms in the RAS and eNOS genes have been extensively studied for their role in CAD susceptibility. In this study, we investigated the association of ACE I/D and eNOS-786 polymorphisms with CAD risk in the Pashtun population of Khyber Pakhtunkhwa, Pakistan. Our findings reveal a significant association between the ACE I/D polymorphism and CAD risk, whereas no such correlation was observed for the eNOS-786 polymorphism. The ACE I/D polymorphism has been widely studied in relation to CVDs, with several studies indicating its role in modulating blood pressure regulation and vascular remodeling.^[14,15]^ Our results demonstrated that the deletion (D) allele was significantly more frequent among CAD patients (75%) compared to controls (40%), with an OR of 4.76 (95% CI:3.21–7.08, *P* < .001). Furthermore, individuals carrying the DD genotype exhibited a markedly higher risk of developing CAD (OR = 6.42, 95% CI:3.89–10.59, *P* < .001) in comparison to those with the II genotype. These findings align with previous studies conducted in different populations, where the DD genotype has been consistently linked to increased cardiovascular risk due to elevated ACE activity, leading to higher angiotensin II levels, vasoconstriction, and subsequent hypertension.^[16,17]^

Conversely, the eNOS-786 polymorphism did not show a significant association with CAD in our study population. The distribution of TT, TC, and CC genotypes was similar between cases and controls, with an OR of 1.08 (95% CI: 0.72–1.61, *P* = .98). This suggests that, at least in the Pashtun population, the eNOS-786 polymorphism does not play a substantial role in modulating CAD risk. Our findings are consistent with several previous studies that failed to establish a definitive link between eNOS polymorphisms and CVDs.^[18–22]^ However, some studies in other ethnic groups have reported significant associations, indicating potential population-specific genetic influences.^[23–25]^ The role of the ACE DD genotype in CAD can be attributed to its influence on the RAS. The presence of the DD genotype has been associated with increased ACE enzyme activity, resulting in higher levels of angiotensin II, a potent vasoconstrictor that contributes to endothelial dysfunction, arterial stiffness, and plaque formation.^[26–28]^ Additionally, the ACE DD genotype has been linked to left ventricular hypertrophy, myocardial infarction, and stroke, further emphasizing its impact on cardiovascular pathology.^[29,30]^

In the study population, individuals with the ACE DD genotype exhibited a higher prevalence of hypertension (55%), type 2 diabetes (50%), and hyperlipidemia (45%), further supporting its role in CAD pathogenesis. These findings are consistent with reports suggesting that the ACE DD genotype is associated with increased blood pressure, insulin resistance and hyperlipidemia all of which are established risk factors for CAD.^[31,32]^ In contrast, no significant association was observed between eNOS-786 polymorphism and these clinical parameters, reinforcing the notion that this polymorphism may have a lesser impact on CAD risk in our study population. Population-specific genetic variations may account for differences in the association of these polymorphisms with CAD across different ethnic groups.^[33–35]^ The Pashtun population is genetically distinct from other South Asian populations/ethinic groups having unique genetic make-up and living habits, which may contribute to variations in gene-disease associations. Further studies involving larger sample sizes and genome-wide approaches are warranted to explore additional genetic determinants influencing CAD risk in this population.

## 5. Study limitations

Despite the significant findings of this study there are few limitations of this study also that is, the present study includes study participants from Pashtun population only whereas other ethnic groups were ignored or not considered. Additionally, the study did not include a family-based analysis, which could have offered deeper insights into the hereditary risk of CAD within the population. Future large scale genomics research/study involving multiple ethnic groups and longitudinal follow-ups will provide deeper insights into the genetic risk variants associated with CAD, understanding the underlying mechanism, early detection and improved management of this life-threatening disease.

## 6. Conclusion

Our study provides compelling evidence for a strong association between the ACE I/D polymorphism and CAD risk in the Pashtun population of Khyber Pakhtunkhwa, Pakistan. The ACE DD genotype was significantly more frequent among CAD patients and was associated with increased odds of disease susceptibility. In contrast, the eNOS-786 polymorphism did not demonstrate a significant correlation with CAD, suggesting that it may not be a major genetic determinant of cardiovascular risk in this population. These findings underscore the potential utility of genetic screening for ACE I/D polymorphism as part of early risk assessment strategies for individuals predisposed to CAD. Given the strong association of the DD genotype with hypertension, diabetes, and hyperlipidemia, targeted interventions such as personalized treatment strategies and lifestyle modifications could be implemented for high-risk individuals. Future research should focus on expanding the genetic investigation of CAD in diverse ethnic groups, incorporating additional polymorphisms and environmental factors. A comprehensive understanding of genetic predisposition to CAD will aid in developing precision medicine approaches tailored to specific populations, ultimately improving cardiovascular health outcomes.

## Author contributions

**Conceptualization:** Asif Jan, Aftab Ullah, Ursula Abu Nahla.

**Data curation:** Asif Jan, Aftab Ullah, Mashal Khattak, Ursula Abu Nahla.

**Formal analysis:** Asif Jan, Mashal Khattak, Ursula Abu Nahla.

**Investigation:** Asif Jan, Mashal Khattak, Rani Akbar.

**Methodology:** Asif Jan, Aftab Ullah, Mashal Khattak, Waheed Ali Shah.

**Supervision:** Ursula Abu Nahla.

**Validation:** Aftab Ullah, Waheed Ali Shah.

**Visualization:** Aftab Ullah, Waheed Ali Shah, Rani Akbar, Ursula Abu Nahla.

**Writing – original draft:** Asif Jan, Rani Akbar.

**Writing – review & editing:** Waheed Ali Shah, Rani Akbar, Ursula Abu Nahla.
